# Hypoalbuminemia affects the spatio-temporal tissue distribution of ochratoxin A in liver and kidneys: consequences for organ toxicity

**DOI:** 10.1007/s00204-022-03361-8

**Published:** 2022-08-13

**Authors:** Reham Hassan, Adrian Friebel, Lisa Brackhagen, Zaynab Hobloss, Maiju Myllys, Daniela González, Wiebke Albrecht, Elsayed S. I. Mohammed, Abdel-latif Seddek, Rosemarie Marchan, Cristina Cadenas, Benedikt Cramer, Hans-Ulrich Humpf, Lukas Hartl, Benedikt Simbrunner, Thomas Reiberger, Michael Trauner, Stefan Hoehme, Gisela H. Degen, Jan G. Hengstler, Ahmed Ghallab

**Affiliations:** 1 Leibniz Research Centre for Working Environment and Human Factors, Technical University Dortmund, Ardeystr. 67, 44139 Dortmund, Germany; 2 Department of Forensic Medicine and Toxicology, Faculty of Veterinary Medicine, South Valley University, Qena, 83523 Egypt; 3 Institute of Computer Science and Saxonian Incubator for Clinical Research (SIKT), University of Leipzig, Haertelstraße 16-18, 04107 Leipzig, Germany; 4 Department of Histology and Cytology, Faculty of Veterinary Medicine, South Valley University, Qena, Egypt; 5 Institute of Food Chemistry, Westfälische Wilhelms-Universität Münster, Corrensstr. 45, 48149 Münster, Germany; 6 Hans Popper Laboratory of Molecular Hepatology, Division of Gastroenterology and Hepatology, Department of Internal Medicine III, Medical University of Vienna, Vienna, Austria; 7 Vienna Hepatic Hemodynamic Lab, Division of Gastroenterology and Hepatology, Department of Internal Medicine III, Medical University of Vienna, Vienna, Austria; 8 Christian Doppler Lab for Portal Hypertension and Liver Fibrosis, Medical University of Vienna, Vienna, Austria

**Keywords:** Intravital imaging, Mycotoxins, Albumin binding, Pharmacokinetics, Toxicokinetics

## Abstract

Hypoalbuminemia (HA) is frequently observed in systemic inflammatory diseases and in liver disease. However, the influence of HA on the pharmacokinetics and toxicity of compounds with high plasma albumin binding remained insufficiently studied. The ‘*lack-of-delivery-concept*’ postulates that HA leads to less carrier mediated uptake of albumin bound substances into hepatocytes and to less glomerular filtration; in contrast, the ‘*concept-of-higher-free-fraction*’ argues that increased concentrations of non-albumin bound compounds facilitate hepatocellular uptake and enhance glomerular filtration. To address this question, we performed intravital imaging on livers and kidneys of anesthetized mice to quantify the spatio-temporal tissue distribution of the mycotoxin ochratoxin A (OTA) based on its auto-fluorescence in albumin knockout and wild-type mice. HA strongly enhanced the uptake of OTA from the sinusoidal blood into hepatocytes, followed by faster secretion into bile canaliculi. These toxicokinetic changes were associated with increased hepatotoxicity in heterozygous albumin knockout mice for which serum albumin was reduced to a similar extent as in patients with severe hypoalbuminemia. HA also led to a shorter half-life of OTA in renal capillaries, increased glomerular filtration, and to enhanced uptake of OTA into tubular epithelial cells. In conclusion, the results favor the ‘*concept-of-higher-free-fraction*’ in HA; accordingly, HA causes an increased tissue uptake of compounds with high albumin binding and increased organ toxicity. It should be studied if this concept can be generalized to all compounds with high plasma albumin binding that are substrates of hepatocyte and renal tubular epithelial cell carriers.

**Supplementary Information:**

The online version contains supplementary material available at 10.1007/s00204-022-03361-8.

## Introduction

Serum albumin binds numerous endogenous and exogenous compounds, including drugs and environmental toxicants (Merlot et al. 2014; Soeters et al. 2019). It is also the most abundant plasma protein with concentrations ranging between 35 and 50 g/liter (Merlot et al. 2014). A strong decrease in serum albumin can be caused by inflammatory diseases, mostly because of increased capillary permeability and leakiness to the interstitial space (Fanali et al. 2012; Soeters et al. 2019). Moreover, hypoalbuminemia is a common feature in patients with liver cirrhosis (Balcar et al. 2021; Hartl et al. 2021a, 2021b) and renal failure (Haller 2005).

Plasma concentrations of albumin strongly influence the pharmacokinetics and toxicity of many compounds (review: (Fanali et al. 2012)). For example, hypoalbuminemic patients showed increased adverse reactions to the anti-epileptic drug phenytoin that extensively binds serum albumin (Vallner 1977). Similarly, aggravated adverse reactions to diazepam were reported in hypoalbuminemia (Greenblatt and Koch-Weser 1974) and low serum albumin was associated with longer methotrexate clearance (Reiss et al. 2016). However, the influence of hypoalbuminemia on pharmacokinetics and toxicity of xenobiotics is complex and incompletely understood (Fanali et al. 2012). Plasma proteins have been reported to serve as delivery systems of compounds to hepatocytes or to glomerular filtration if the protein-bound compounds are substrates of active transport mechanisms in these tissues (Fanali et al. 2012). Under these circumstances, low plasma proteins would decrease hepatic/renal uptake of drugs (Fanali et al. 2012).

To date, research on the influence of hypoalbuminemia on pharmacokinetics/toxicokinetics has been hampered by the lack of techniques that permit the direct observation of compound transport from blood to specific cell types. To address this aspect, we used a two-photon based intravital imaging technique that allows for the spatio-temporal observation of compound transport from the blood capillaries (sinusoids) of the liver into the hepatocytes followed by secretion into bile canaliculi (Ghallab et al. 2019a). These analyses can be performed in intact livers of anesthetized mice (Remetic et al. 2022). Moreover, compound filtration from renal capillaries into Bowman’s space, and the further transport into the lumen of tubules and subsequent uptake into the cytoplasm of tubular epithelial cells can be analyzed by this technique (Ghallab et al. 2021a; Koeppert et al. 2021; Reif et al. 2017).

Ochratoxin A (OTA), a mycotoxin of relevance for human health (EFSA 2020), has a high plasma protein-binding capacity (> 99%); it is actively taken up from the sinusoidal blood into hepatocytes by organic anion-transporting polypeptides (OATPs), and then actively exported from hepatocytes into bile canaliculi by multidrug resistance-associated protein 2 (MRP2 or ABCC2) and breast cancer resistance protein (BCRP) (Table 1) (Anzai et al. 2010; Ringot et al. 2006). Based on its auto-fluorescence properties, label-free intravital imaging of OTA has already been established (Ghallab et al. 2021a), which permits us to re-evaluate two already-published concepts: (1) “If a drug is highly bound to plasma proteins and has a high extraction ratio by the liver, which involves active transport mechanisms to concentrate the drug in the hepatocytes, then plasma proteins act as delivery systems” (Fanali et al. 2012), and (2) “If the protein-bound drug cannot readily leave the capillaries, only the unbound drug can be distributed to tissues, therefore having pharmacological activity, as well as toxic effects” (Fanali et al. 2012). Following up of these two concepts may lead to contradicting conclusions. According to the first, hypoalbuminemia should lead to lower concentrations of OTA in hepatocytes, because of the lack of albumin, which functions as a delivery system (‘lack of delivery concept’). The second concept suggests that hypoalbuminemia leads to an increased non-protein-bound fraction of OTA and therefore to higher concentrations of OTA in hepatocytes (‘concept of higher free fraction’). Based on theoretical considerations only, it is difficult to determine which concept is correct. The ‘lack of delivery concept’ depends on the assumption that the hepatocyte uptake carriers for OTA (the OATPs) can accept the OTA ‘delivered’ by albumin as a substrate; alternatively, OTA may be bound so tightly to albumin that it is not available for uptake carriers. If the latter ‘concept of higher free fraction’ is correct, then the distribution volume of OTA in hypoalbuminemia should strongly increase.

**Table 1:** Physicochemical properties, half-life, transporters, and toxicity of ochratoxin A

Compound	Ochratoxin A (OTA)		References
Structure			(EFSA 2020)
CAS-number	303–47-9		
Molecular formula	C_20_H_18_ClNO_6_		
Molecular mass (g/mol)	403.8		
Log Pow	4.37		
pKa values	4.3 and 7.2 for the carboxyl and the phenolic hydroxyl group (weak organic acid)		(Perry et al. 2003)
Albumin binding	HSA: K ~ 10^7^ (log K 7.0 – 7.6)		(Poor et al. 2013; Sueck et al. 2018)
Half-life in blood	Mice: t_1/2_ 40 h (*per os*) Human: t_1/2_ 35.5 days		(O'Brien and Dietrich 2005; Ringot et al. 2006)
Transporters	Liver	Basolateral: OATPs	(Anzai et al. 2010; George et al. 2017; Kontaxi et al. 1996)
		Apical: Mrp2, BCRP	
	Kidney	Basal: OATs	
		Apical: Mrp2, BCRP, NPT4	
Acute toxicity (LD_50_) in mice	Range (*i.v.)* 25.7 – 33.8 mg/kg Range (*p.o.)* 46 – 58.3 mg/kg		(IARC 1993)

We addressed the above-described hypotheses by intravital imaging of OTA transport in albumin knockout mice, in which serum albumin concentrations are reduced to the levels of patients with severe hypoalbuminemia in heterozygous mice and below the detection limit in homozygous animals. The results demonstrate that reduced serum albumin concentrations strongly increase the glomerular filtration of OTA, as well as its uptake from the sinusoidal blood into hepatocytes resulting in increased hepatotoxicity, and therefore clearly support the ‘concept of higher free fraction’ rather than the ‘lack of delivery concept’.

## Materials and methods

### Patients

Serum albumin data from two cohorts of patients with advanced chronic liver disease (ACLD) were analyzed, one cohort with 663 ACLD patients (Hartl et al. 2021a) and a second cohort with 168 ACLD patients (Costa et al. 2021). Albumin concentrations of patients with ACLD were normalized to data of 1812 individuals (never smokers) randomly drawn from one general practice in each of 24 towns in England, Wales, and Scotland (Phillips et al. 1989).

### Mice

Six-to-eight-week-old albumin knockout C57BL/6 J-Alb^em8Mvw/MvwJ^ (#025,200; The Jackson Laboratory) and corresponding C57BL/6 J wild-type (Janvier Labs, France) mice were used. Based on genotyping results, both homozygous (albumin^−/−^) and heterozygous (albumin^±^) albumin-deficient mice were included in the study. The mice were fed ad libitum on a regular chow diet (Ssniff) with free access to drinking water and were housed under standard light and temperature conditions. All experiments were approved by the local animal welfare committee (#81–02.04.2020.A304; LANUV, North Rhine-Westphalia, Germany).

### Ochratoxin A toxicity experiment

To study the role of albumin in OTA toxicity, 8-week-old female homozygote and heterozygote albumin-deficient mice, and corresponding C57BL/6 J wild-type controls were challenged intravenously with 10 mg/kg OTA (#10470691, Fisher Scientific) dissolved in 0.1 M NaHCO_3_, pH 7.4, with an application volume of 2 ml/kg. On day 1 after OTA injection, heart blood samples were collected, and plasma was separated according to a standard protocol (Gianmoena et al. 2021). In addition, tissue specimens were collected from the left liver lobe and kidneys, fixed for 2 days in 4% paraformaldehyde (#P087-5, Roth), and finally embedded in paraffin for histopathology and immunohistochemistry analyses (Schneider et al. 2021b).

### Biochemical analysis of mouse plasma

Plasma concentration of albumin, creatinine, and blood urea nitrogen (BUN), as well as activities of alanine transaminase (ALT), and aspartate transaminase (AST) were analyzed by the Piccolo Xpress Chemistry Analyzer (Hitado, Germany) using the Piccolo general chemistry 13 panel (#AB-114-400-0029, Hitado, Germany).

### Histopathology

Hematoxylin and eosin (H&E) staining was performed in 4 µm-thick paraffin-embedded liver and kidney tissue sections as previously described (Campos et al. 2020).

### Immunostaining

Visualization of albumin protein expression in the liver tissue, and immune cell infiltration in the liver and kidney tissues was done by immunohistochemistry using an autostainer (Discovery Ultra Automated Slide Preparation System, Roche, Germany) (Ghallab et al. 2021b). For this purpose, 4 µm-thick paraffin-embedded liver or kidney tissue sections were incubated with primary antibodies against albumin (# ab192603, abcam), CD45 (a pan leukocyte marker; #550,539, BD Bioscience), and FSP1 (a marker of inflammatory subpopulation of macrophages (Österreicher et al. 2011) (#ab197896, Abcam). Dilutions of the primary antibodies as well as the used secondary antibodies are described in Table 2. Following staining, entire slides were scanned (Axio Scan.Z1, Zeiss, Germany) and representative images are shown in the results section.

**Table 2:** Setup for immunohistochemistry and intravital imaging

Antibodies used for immunostaining				
Target	Primary antibodies		Secondary antibodies	
	Antibody	Dilution	Antibody	Dilution
Albumin	Anti-albumin, rabbit	1:500	Ultra-Map anti-rabbit HRP	Automatic Discovery Ready to use
Leukocytes	Anti-mouse CD45, rat	1:400	Ultra-Map anti-rat HRP	
Inflammatory subpopulation of macrophages	Anti-S100A4, rabbit	1:200	Ultra-Map anti-rabbit HRP	

Intravital imaging conditions of ochratoxin A and TMRE				
Dye/toxin	Marker for	Dose [mg/kg]	Vehicle	Excitation range [nm]
TMRE	Mitochondrial membrane potential	0.96	Methanol/PBS (1:1)	780–820
Ochratoxin A	Ochratoxin A	5	0.1 M NaHCO_3_	740–780

### Intravital imaging of ochratoxin A transport in the liver and kidneys

Intravital imaging of OTA transport in the livers and the kidneys of anesthetized wild-type and albumin knockout mice was done using an inverted two-photon microscope (LSM MP7, Zeiss, Germany). Details of the surgical preparation of the mice and exposure of the liver and the kidneys for imaging were previously described (Ghallab et al. 2021a; Reif et al. 2017). Tissue morphology was visualized by intravenous administration of the mitochondrial membrane potential marker tetramethylrhodamine ethyl ester (TMRE) (#T669, ThermoFisher Scientific) approximately 10 min before recording (Table 2). To image its transport kinetics in the liver and kidneys, a bolus of OTA (5 mg/kg) was administered via a tail vein catheter (SAI-infusion, IL, USA) within approximately 20 s after the onset of recording; the first few frames recorded prior to the OTA administration served to detect the basal tissue auto-fluorescence (Table 2). The mice were kept under controlled ambient temperature conditions of 36 °C, with exposure to a maintenance isoflurane inhalation anesthesia during the entire recording session.

### Image analysis

During image processing, rigid body registration was performed using StackReg (Thévenaz et al. 1998) to compensate for tissue movement in the recorded time series. From these stabilized videos, 2D projections were generated using average and standard deviation operators. The pixel classification and autocontext workflows of the interactive image segmentation software ilastik (version 1.3.3post1) (Berg et al. 2019) were used for compartment segmentation in the 2D projections of liver and kidney. The compartments considered are sinusoids, hepatocyte cytoplasm and bile canaliculi in the liver, as well as inter-tubular capillaries, renal tubular lumen, and the tubular epithelial cells in the kidney. Mean raw OTA intensities were measured per compartment and frame. In addition, for the kidney, mean OTA intensities per tubule were measured based on the signal in the TMRE positive cell compartment, and tubules were subsequently grouped into two sets using k-means clustering based on their maximum mean OTA intensity over time.

### Calculation of elimination half-life

For the calculation of the elimination half-life, Microsoft excel was used. First, the decadic logarithm of the intensity was plotted against the time. Then, the linear declining portion of the curve was selected manually and used for construction of a trend line. Next, the slope of the trendline was multiplied by 2.303 to convert the decadic to the natural logarithm and enable usage of the following formulas:

1. *kel* = *-slope (Certara knowledgebase)*
2. *T ½* = *ln (2) /kel (Certara knowledgebase)*

(https://www.certara.com/knowledge-base).

### Statistical analysis

Data analysis was done using GraphPad Prism version 9.3.1 (GraphPad Software, Inc., La Jolla, CA). Significance level is indicated as *p value ≤ 0.05, **p value ≤ 0.01, ***p value ≤ 0.001 using Dunnett's or Tukey's multiple comparisons test.

## Results

### Characterization of the albumin knockout mouse model and translational relevance

**Fig. 1:**
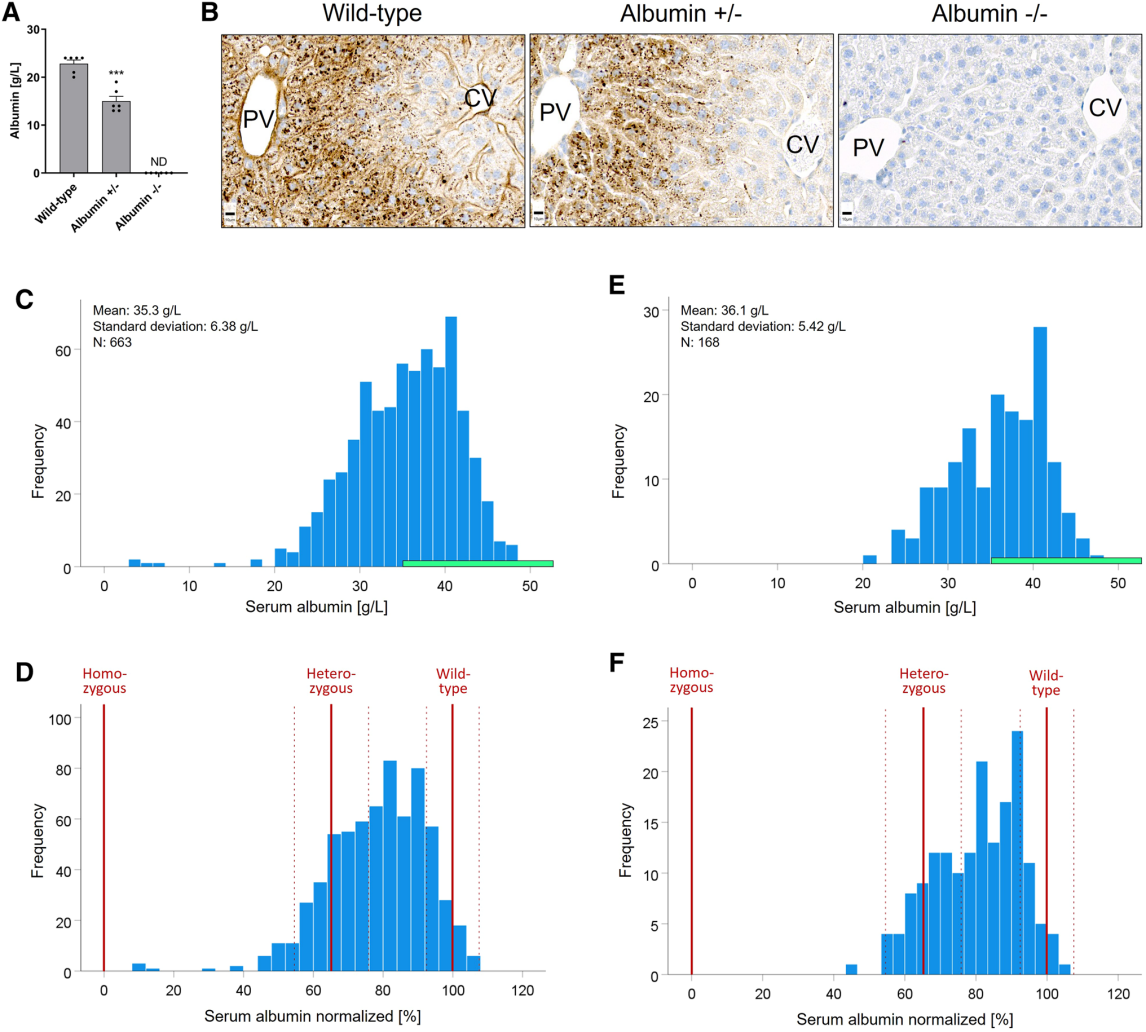
Plasma albumin concentrations in albumin knockout mice compared to patients with chronic liver diseases. **A** Albumin concentrations in wild-type, heterozygous (albumin ±), and homozygous (albumin −/−) mice. Data are means and standard errors of 6 mice per group. ***p value ≤ 0.001 compared to wild-type mice using Dunnett's multiple comparisons test. **B** Albumin immunostaining in wild-type and albumin knockout mice; Scale bars: 10 µm; PV: portal vein; CV: central vein. C–F Serum albumin levels in albumin knockout mice in relation to patients with advanced chronic liver disease (ACLD). **C** Cohort of 663 patients with advanced chronic liver disease. The green bar indicates the normal range of 35–55 g/L. **D** Normalized serum albumin of the same cohort, whereby 45 g/L were considered as 100%. The red vertical bars show normalized serum albumin of the homozygous, heterozygous, and wild-type mice (solid line: mean; dashed line: standard deviation of the mice shown in **A**). **E** and **F** show corresponding analyses of a second previously published study with serum albumin data of 168 patients with advanced chronic liver disease (colour figure online)

To characterize the albumin knockout mice, serum albumin levels were determined, resulting in means of 23 and 15 g/L in wild-type and heterozygous mice, respectively (Fig. 1A). In homozygous animals, serum albumin was below the detection limit. Moreover, albumin protein expression in hepatocytes was analyzed by immunohistochemistry. The albumin signal showed a zonated expression pattern predominantly in the periportal hepatocytes in wild-type mice (Fig. 1B). In heterozygous albumin knockout mice, albumin expression was similarly zonated in the liver lobule as in the wild-type mice but with a reduced signal (Fig. B). In contrast, albumin was not detectable in any hepatocytes of the homozygous albumin knockout mice (Fig. 1B). To estimate the clinical relevance of the data obtained from the albumin knockout mice, their albumin blood concentrations were compared to those of patients with liver disease (Fig. 1C–F). In a cohort of 663 patients with advanced chronic liver disease (Hartl et al. 2021a, b), the mean albumin concentration was at 35.3 g/L, which are considerably lower compared to values of randomly drawn individuals (45.0 g/L in non-smokers; 44.0 g/L in heavy smokers (Phillips et al. 1989). Notably, 25% and 5% of the ACLD patients showed albumin levels < 31.2 g/L and < 24.9 g/L, respectively (Fig. 1C). Since mean albumin levels in humans are higher compared to wild-type mice, we normalized the data, so that 100% corresponds to 45 g/L in humans and 23 g/L in mice, respectively. Comparing normalized mouse and human serum albumin concentrations showed that the heterozygous albumin knockout mice ranged into the lowest quartile of the patients, while the homozygous animals were below the patients with the lowest serum albumin concentrations in this cohort (Fig. 1D). A similar analysis was performed for a second dataset derived from a previously published cohort of 168 ACLD patients with available serum albumin levels (Costa et al. 2021). The mean albumin levels in this second cohort of ACLD patients were again low at 36.1 g/L, with a considerable proportion of patients showing albumin levels in the reduced range observed in heterozygous knockout mice—confirming that indeed, HA occurs to a similar degree as in the animal models in patients with liver disease (Fig. 1E, F).

### Hypoalbuminemia enhances hepatocellular uptake and biliary excretion of OTA

**Fig. 2:**
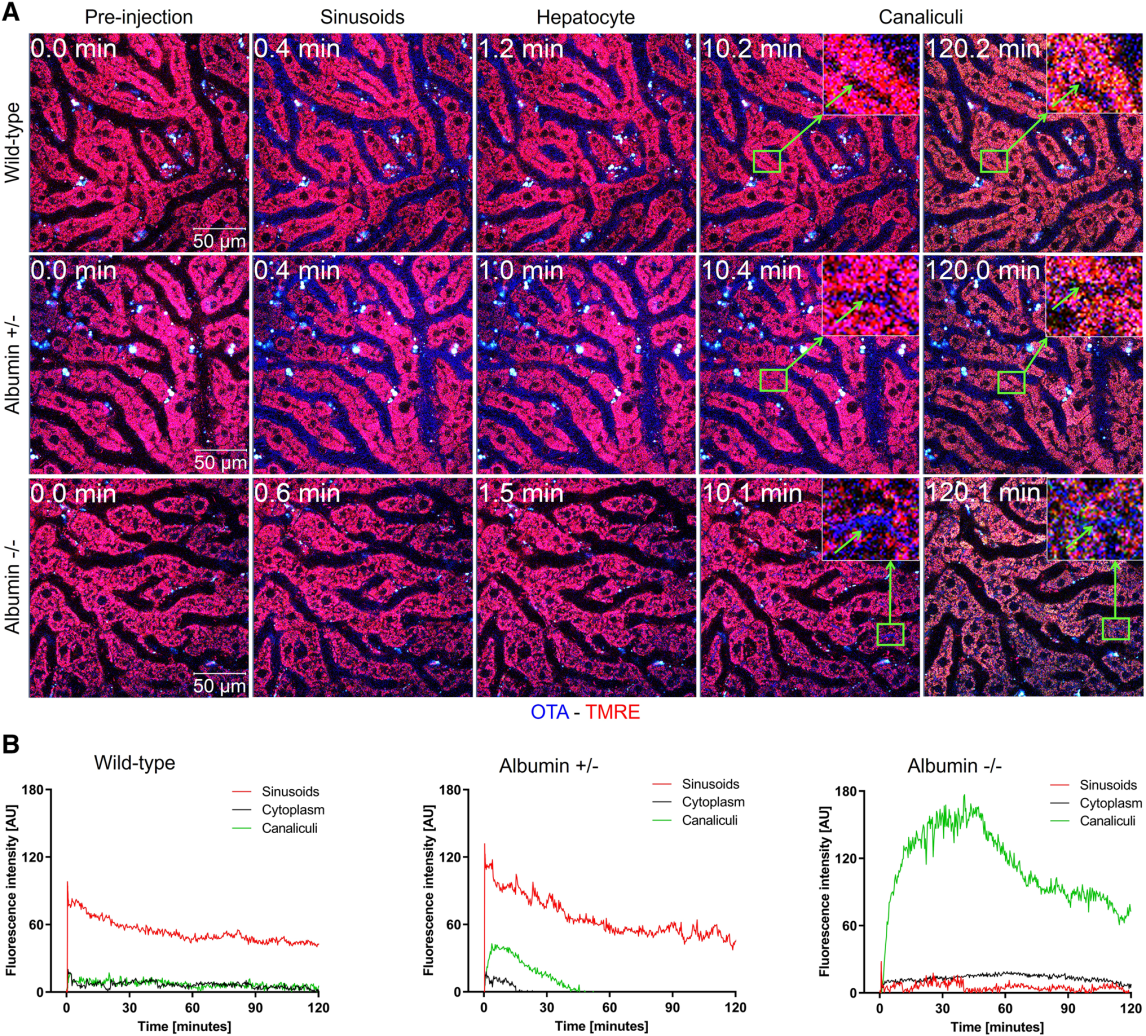
Intravital imaging of OTA transport in the liver. Decreased half-life of OTA in sinusoidal blood, increased intensity in hepatocytes, and increased bile canalicular secretion due to the albumin knockout. **A** Two-photon imaging of the liver of wild-type, heterozygous (albumin ±), and homozygous (albumin −/−) albumin knockout mice. The time after tail vein bolus injection of 5 mg/kg OTA is given in the upper left corner. The blue OTA-associated signal initially occurs in the blood sinusoids, followed by hepatocyte uptake and bile canalicular secretion. Scale bars: 50 µm; the stills correspond to Supplementary Video 1A-C. **B** Quantification of the OTA signal in sinusoids, hepatocyte cytoplasm, and bile canaliculi of the three mouse models. The images and quantifications are representative of 3 mice per condition

To study the influence of albumin on the transport of OTA from the sinusoidal blood into hepatocytes, intravital imaging was performed on anesthetized mice after tail vein injection of 5 mg/kg OTA (Fig. 2; Supplementary Video 1). The intravital videos of wild-type mice showed a sharp increase in the OTA signal in sinusoidal blood within seconds after injection; after an initial decrease, probably due to redistribution, a plateau was obtained. No transport of OTA to hepatocytes and bile canaliculi was detectable (Fig. 2B; Supplementary Video 1A). The situation was clearly different in the heterozygous mice where the increase of the OTA signal in the sinusoidal blood was followed by transient increases in hepatocytes and enrichment in bile canaliculi (Fig. 2B; Supplementary Video 1B). A completely different scenario occurred in the homozygous albumin knockout mice where the OTA signal in blood rapidly decreased, with a short half-life of approximately 0.22 min, followed by massively increased canalicular secretion with almost no increase in the hepatocytes (Fig. 2B; Supplementary Video 1C). Thus, hypoalbuminemia enhanced the hepatocyte uptake and the biliary excretion of OTA.

### Hypoalbuminemia enhances glomerular filtration of OTA and uptake into tubular epithelial cells

**Fig. 3:**
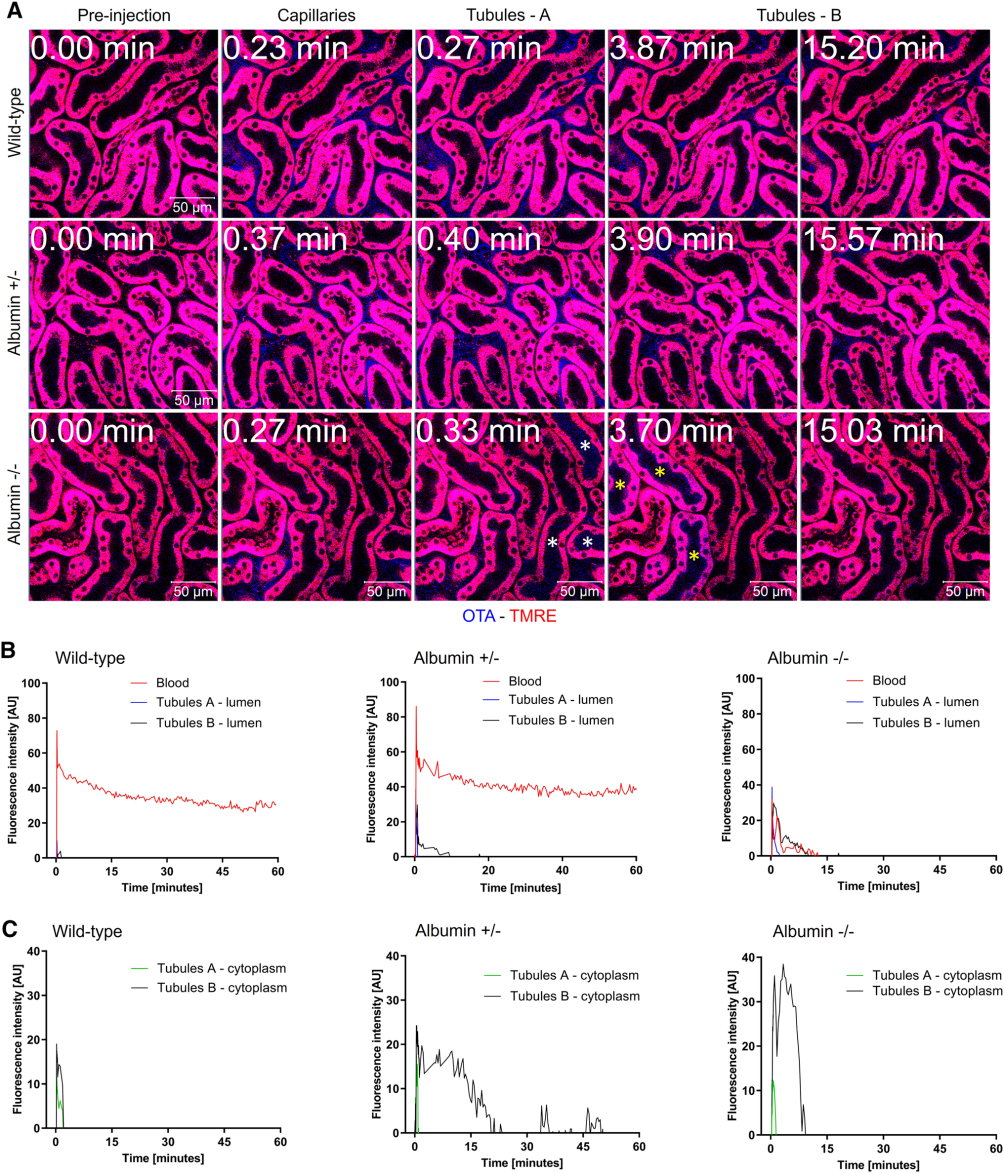
Intravital imaging of OTA transport in the kidney. Decreased half-life of OTA in blood capillaries, filtration into the tubular lumen, and enrichment in tubular epithelial cells due to the albumin knockout. **A** Two-photon imaging of the kidney of wild-type, heterozygous (albumin ±), and homozygous (albumin −/−) albumin knockout mice. The time after tail vein bolus injection of 5 mg/kg OTA is given in the upper left corner. The blue OTA-associated signal initially occurs in the inter-tubular capillaries and later in the lumen of tubules and in tubular epithelial cells. The white and the yellow asterisks in the images of homozygous mouse represent tubules A and B, respectively. Scale bars: 50 µm; the stills correspond to Supplementary Video 2A-C. B, C. Quantification of the OTA signal in blood of inter-tubular capillaries, tubular lumen (**B**) and tubular epithelial cells (**C**) of the three mouse models. The images and quantifications are representative of 3 mice per condition

Intravital imaging of the kidneys was performed to quantify the OTA signal in the renal blood capillaries, tubular lumen, and in the tubular epithelial cells (Fig. 3A; Supplementary Video 2). After bolus tail vein injection of 5 mg/kg OTA, we observed an initial sharp increase in the renal blood capillaries and redistribution followed by a plateau in the wild-type mice (Fig. 3B), similar as in the sinusoids of the liver (Fig. 2B). Based on the time of appearance of the OTA signal, two sets of renal tubules could be differentiated: tubules A, included those with early appearance of the OTA signal in their lumen; and tubules B, included those with later appearance of the OTA signal in their lumen; this was clearly visible in the homozygous mice. In wild-type mice, only a weak, transient increase was seen in the lumen and cytoplasm of the tubular epithelial cells of both tubule sets (Fig. 3A–C; Supplementary Video 2A). Imaging of the heterozygous mice showed filtration into the tubular lumens (Fig. 3B) and a longer duration and higher intensity of the OTA signal in the tubular epithelial cells (Fig. 3A–C; Supplementary Video 2B). Concerning the intracellular OTA signal, tubules A showed a narrow initial peak that coincided with the increase of their luminal signal. In contrast, tubules B showed a strong and longer lasting increase. In the homozygous knockout mice, the OTA signal in the blood capillaries rapidly decreased after an initial inflow with a half-life of approximately 0.28 min (Fig. 3B), similar to the situation in the sinusoids of the liver (Fig. 2B). This coincided with rapid filtration into the renal tubular lumen and enrichment into the tubular epithelial cells, particularly in the cytoplasm of tubules B (Fig. 3A–C; Supplementary Video 2C).

### Increased hepatotoxicity of OTA in albumin knockout mice

**Fig. 4:**
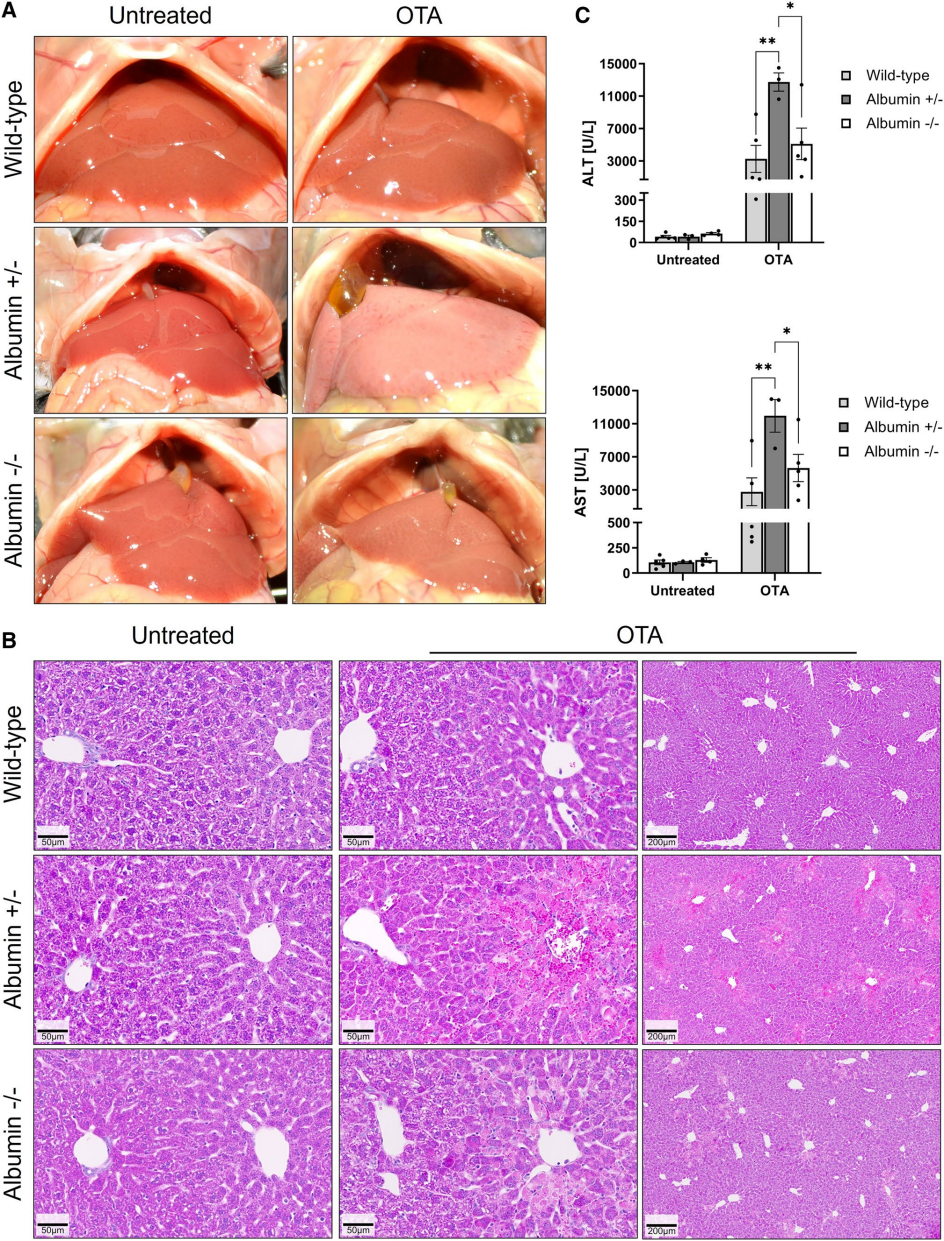
Increased hepatotoxicity of OTA in heterozygous albumin knockout mice. **A** Gross pathology. **B** Histopathology with hematoxylin and eosin staining; scale bars: 50 µm (untreated and left panel of OTA) and 200 µm (right panel of OTA). **C** Liver enzyme activities in the blood of untreated and OTA-treated wild-type, heterozygous (albumin ±) and homozygous (albumin −/−) albumin knockout mice. *p value ≤ 0.05, **p value ≤ 0.01 Tukey's multiple comparisons test; *n* = 5 mice per group

**Fig. 5:**
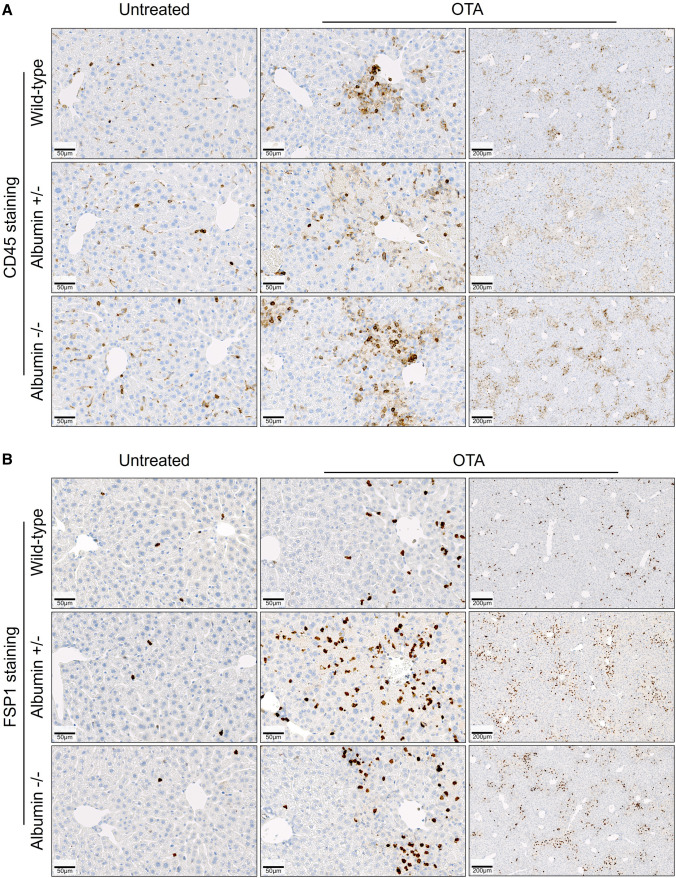
Increased immune cell infiltration into the pericentral compartment of the liver lobule after OTA intoxication. **A** Immunostaining of CD45, a pan marker of leukocytes. **B** Immunostaining of FSP1, a marker of inflammatory subpopulation of macrophages. Liver tissue was analyzed 24 h after tail vein injection of 10 mg/kg OTA. Scale bars: 50 µm (untreated and left panel of OTA) and 200 µm (right panel of OTA). Representative images of 5 mice per group are shown

To investigate if the concentration of serum albumin influences OTA-mediated hepato- or nephrotoxicity, a single dose of 10 mg/kg OTA was intravenously administered to wild-type, heterozygous and homozygous albumin knockout mice and the blood, as well as liver and kidney tissues were analyzed 24 h after administration. No differences in macroscopical (Fig. 4A) and histological (Fig. 4B) appearance between livers of untreated wild-type, heterozygous as well as homozygous albumin knockout mice were observed. Macroscopically, the livers of treated heterozygous mice appeared paler compared to wild-type and homozygous animals (Fig. 4A). Histopathological analysis showed small clusters of dead cells in the pericentral compartment of the liver lobule after administration of OTA into the wild-type mice (Fig. 4B, upper panel). In contrast, massive pericentral necrosis and hemorrhage was observed in the heterozygous albumin knockout mice after OTA administration (Fig. 4B, middle panel). Surprisingly, although they occurred slightly more frequently than in the wild-type mice, the necrotic regions in the homozygous albumin knockout mice appeared smaller compared to the heterozygous animals (Fig. 4C, lower panel). In agreement with the histological findings, analysis of the blood biomarkers of liver injury ALT and AST showed significantly increased values in the heterozygous albumin-deficient mice compared to the wild-type and homozygous animals (Fig. 4C). To visualize the infiltration of immune cells, stainings with antibodies directed against the pan leukocyte marker CD45 and FSP1, a marker of an inflammatory subpopulation of macrophages, were performed (Fig. 5). Both CD45 (Fig. 5A) and FSP1 (Fig. 5B) showed a pericentral staining pattern, similar to the pericentral necrotic lesions shown in Fig. 4B. Taken together, hypoalbuminemia aggravates OTA hepatotoxicity with a zonated damage pattern in the pericentral compartment of the liver lobule.

**Fig. 6:**
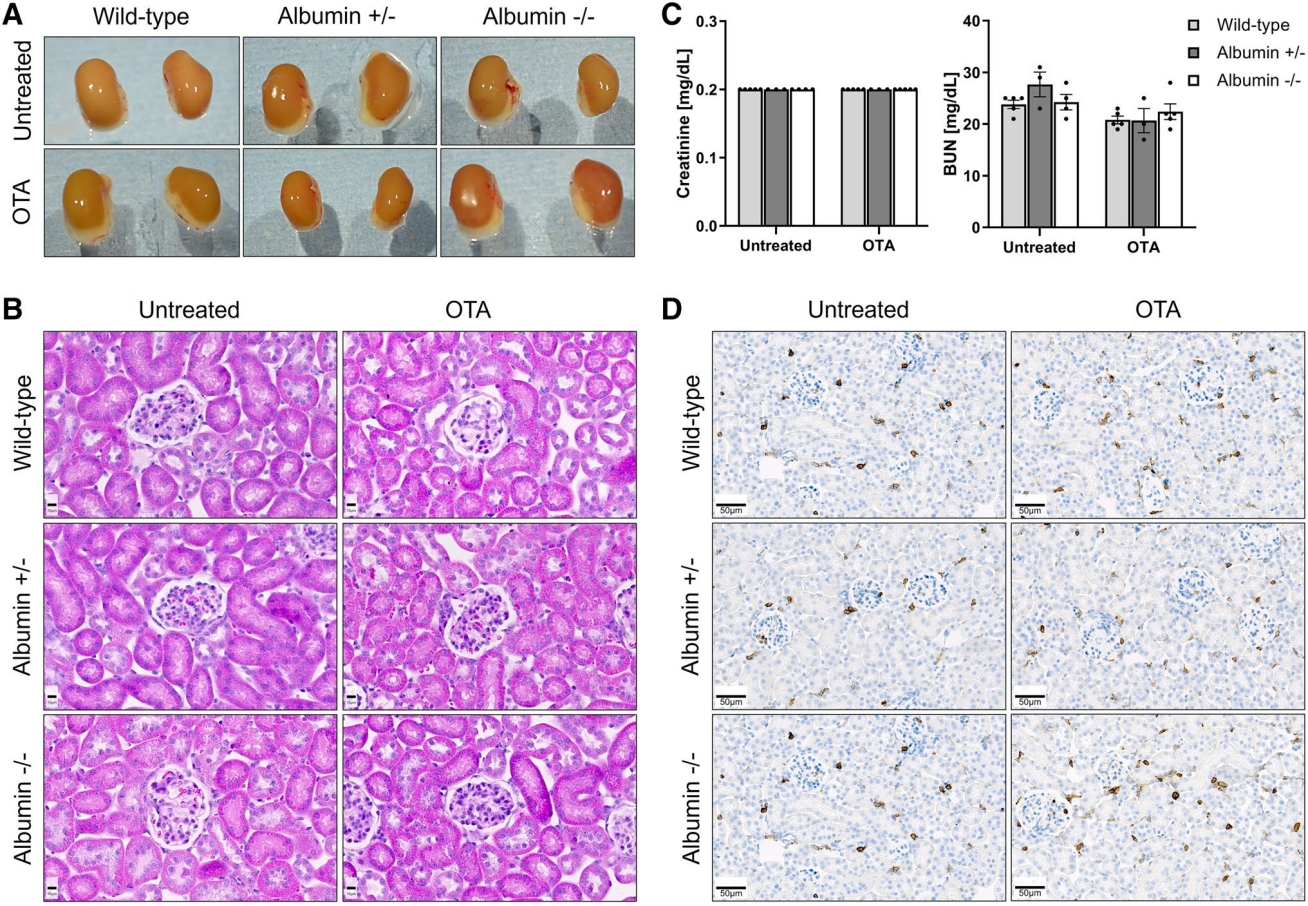
Macroscopic appearance, histology, and clinical chemistry of kidney tissue. Gross pathology (**A**) and histopathology (**B**) with hematoxylin and eosin staining of tissue sections of the kidneys of wild-type, heterozygous, and homozygous albumin knockout mice 24 h after OTA or vehicle treatment. Scale bars: 10 µm. **C** Plasma concentrations of creatinine and urea nitrogen in untreated and OTA intoxicated wild-type and albumin knockout (heterozygous and homozygous) mice; *n* = 5 mice per group. **D** CD45 immunostaining in the kidney tissue showing no obvious immune cell infiltration after OTA administration neither in the wild-type nor in the albumin knockout mice; scale bars: 50 µm. Representative images of 5 mice per group are shown

In contrast to the liver, a single dose of OTA did not cause obvious macroscopical or histopathological alterations in the kidney tissue, neither in wild-type, heterozygous nor homozygous knockout mice (Fig. 6A, B). In agreement, no significant differences in the levels of the blood biomarkers of kidney damage, creatinine and urea nitrogen, were detected between the three mouse models (Fig. 6C). Moreover, no obvious immune cell infiltration was observed after OTA administration neither in the wild type nor in the albumin knockout mice (Fig. 6D).

## Discussion

Recently, techniques in functional intravital imaging have been established that allow for the analysis of the transport of small molecules through tissues (Ghallab et al. 2022; Hassan 2016; Schneider et al. 2021a). Here, we used two-photon imaging to study the influence of hypoalbuminemia on the trafficking of the mycotoxin OTA through tissue compartments of the liver and kidneys. Heterozygous albumin knockout mice, where serum albumin is reduced to levels measured in patients with severe hypoalbuminemia, showed a markedly reduced OTA signal in blood compared to the wild-type mice. This coincided with transiently increased OTA signal in hepatocytes and increased bile canalicular secretion. A probable explanation for this scenario is that decreased plasma albumin leads to higher concentrations of free (non-albumin bound) OTA, which is then available for uptake carriers on hepatocytes, such as OATPs (Kontaxi et al. 1996). Once in the hepatocyte, OTA is rapidly secreted into bile canaliculi by MRP2 and BCRP (Anzai et al. 2010; George et al. 2017) from where it diffuses to the larger ducts, and is further transported by a flow-augmented process to the duodenum (Vartak et al. 2021). An important question is if this relatively rapid passage of OTA through hepatocytes can cause hepatotoxicity. Our results clearly show that reduced serum albumin in the heterozygous knockout mice and the resulting transient increase in hepatocellular OTA concentration lead to enhanced liver damage as evidenced by histological analysis and higher activities of the liver enzymes ALT and AST in blood.

The second scenario—homozygous knockout mice with no serum albumin—is of limited clinical relevance, because to our knowledge, the total absence of albumin has never been reported in patients. Nevertheless, analysis of zero albumin mice is important for a basic understanding of the underlying mechanisms by which albumin influences OTA toxicity. Interestingly, in contrast to the wild-type and heterozygous mice, OTA completely disappeared from the blood of homozygous albumin knockout mice within a minute after bolus injection, which is in agreement with a previous study reporting fast clearance of OTA from the blood of albumin-deficient rats into bile and urine (Kumagai 1985). This indicates that other plasma proteins do not play a major role in OTA transport. A further dramatic difference is the fast and intensive bile canalicular secretion and the surprisingly low OTA concentration in hepatocytes. The latter observation may be explained by the fact that albumin is not only absent in the blood, but also in the hepatocytes, so that albumin-mediated intracellular retention of OTA is lost. Thus, the rate of canalicular secretion by export carriers may be particularly high, and this very fast canalicular secretion of OTA may also explain why homozygous mice experience less toxicity than heterozygous mice.

An unexpected observation was that OTA induced zonated liver damage in the pericentral region of the liver lobules. This phenomenon is known from hepatotoxic compounds that require metabolic activation by cytochrome P450 enzymes, such as acetaminophen (Sezgin et al. 2018) or CCl_4_ (Holland et al. 2022; Schenk et al. 2017), since the activity of cytochrome P450 is higher in the pericentral lobular zone (Ghallab et al. 2019b). OTA is glucuronidated and undergoes hydrolysis to OT-alpha as the major metabolite, whereas the hydroxylated forms 10-OH-OTA, 4(R)- and 4(S)-OH-OTA are minor metabolites of cytochrome P450 enzymes and less toxic than OTA itself (Duarte et al. 2011; Muñoz et al. 2017; Ringot et al. 2006). The recently identified metabolite ochratoxin-N-acetyl-*L*-cystein (OTB-NAC) indicates the formation of glutathione conjugates of OTA, either directly or via a reactive quinone intermediate (Sueck et al. 2020). Nevertheless, it is commonly believed that OTA itself represents the toxic principle (EFSA 2020; Mally and Dekant 2009). While OTA-induced oxidative stress has an important role in vitro, the oxidative stress response is controversial in some in vivo studies (reviewed by (Tao et al. 2018). Considering the preferential damage observed to the cytochrome P450 positive pericentral lobular zone, it may be important to reassess if OTA is metabolically activated to a compound that is more toxic than the parent molecule. A further interesting observation is the zonated expression pattern of albumin with a predominant signal in the periportal hepatocytes. This may contribute to the enhanced toxicity of OTA in the pericentral hepatocytes where albumin expression is very low, so that a higher free fraction of OTA can be expected. However, OTA-induced cell death is also pericentrally zonated in the homozygous albumin knockout mice, although albumin is absent in all hepatocytes. This favors the hypothesis that cytochrome P450-mediated metabolic activation of OTA may be responsible for the zonated pericentral damage.

In the kidney, albumin knockout reduced the half-life of OTA in the blood of capillaries, similar to what was observed in the liver sinusoids. The data are also in agreement with the concept that less albumin in blood leads to a higher fraction of free OTA and, consequently, higher rates of glomerular filtration. From the tubular lumen, OTA is taken up into the tubular epithelial cells by carriers, such as OAT4, Oatp1a1, and OATK1 (George et al. 2017). Therefore, it is plausible that the higher concentrations in the tubular epithelial cells in hypoalbuminemia are a result of enhanced glomerular filtration of OTA and the resulting increased concentration in the tubular lumen. Interestingly, we observed differences between two types of tubular epithelial cells with respect to OTA uptake. ‘Tubules A’ showed only very little uptake, in contrast to ‘tubules B’ with much higher intracellular concentrations. In addition, the OTA signal occurs earlier in the lumen of tubules A than B, leading to the conclusion that tubules A are located more proximal and tubules B more distal to glomeruli. However, we have not yet studied if tubules A and B correspond to proximal and distal tubules, respectively. For this purpose, intravital imaging of OTA with cell type-specific reporter mice of proximal and distal tubules is currently being conducted. In the present study, no obvious nephrotoxicity was observed; however, it should be considered that we only administered a single dose of OTA and the analytical program did not include sensitive readouts of renal toxicity, since we focused on spatial toxicokinetics.

A limitation of the present study is that we only applied intravenous bolus injections of OTA. This route of administration was chosen, because the sudden increase of OTA in the capillaries facilitates the tracking of the compound through different tissue compartments. However, for regulatory purposes, studies with oral administration would be preferred, and future studies should be performed using sub-chronic or chronic oral doses to investigate the degree to which hypoalbuminemia increases OTA toxicity. The LD_50_ of OTA in mice ranges between 25.7 and 33.8 mg/kg (intravenous) and 46 and 58.3 mg/kg (oral) (IARC 1993), and acute toxicity varies between species (O'Brien and Dietrich 2005). In the present study, we have used doses that were clearly lower than the LD_50_ in mice but indeed high compared to human exposure. The doses used here to investigate the disposition of OTA and its toxicity upon single application to mice with different albumin levels are similar as those used in chronic toxicity studies in mice-fed diets containing 40 ppm of the mycotoxin, equivalent to an intake of 5.6 mg/kg b.w. per day that induced liver and kidney tumors (Bendele et al. 1985; Kanisawa and Suzuki 1978).

**Fig. 7:**
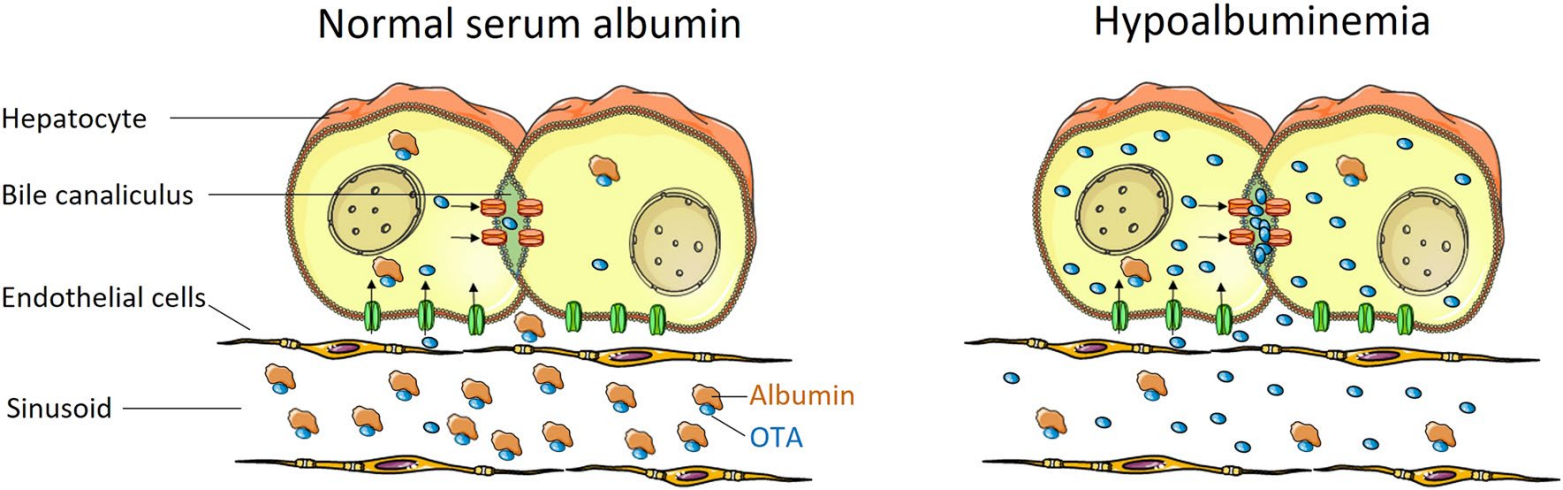
Concept of higher compound uptake due to a higher free fraction. At normal concentrations of serum albumin, a high fraction of OTA is tightly bound to serum albumin and, therefore, not available to carriers that transport OTA from the sinusoidal blood into hepatocytes and further into bile canaliculi. In hypoalbuminemia, the free fraction of OTA is higher. Therefore, more OTA is transported into the hepatocytes. A similar principle applies to tubular epithelial cells of the kidney, where a higher free fraction of OTA is filtered by the glomerulus and is available for active transport into the tubular epithelial cells.

The present study conclusively answers whether the ‘lack of delivery concept’ or the ‘concept of higher free fraction’ applies for OTA. The ‘lack of delivery concept’ was falsified, since hypoalbuminemia led to an increased hepatocellular uptake of OTA. The current data support the ‘concept of higher free fraction’, where decreased serum albumin leads to a higher fraction of non-protein-bound OTA in the blood, which explains the higher uptake into hepatocytes and higher glomerular filtration rate (Fig. 7). It will be interesting to investigate if this concept can be generalized to other substances with high albumin binding and active hepatocellular uptake by carriers. A further consequence of hypoalbuminemia was the faster clearance of OTA from hepatocytes by canalicular secretion, which may be explained by reduced albumin-mediated intracellular retention of OTA. This mechanism is important to interpret the otherwise counterintuitive observation that OTA induces higher levels of hepatotoxicity in heterozygous than in homozygous albumin knockout mice; if intracellular OTA concentrations are very low because of rapid canalicular secretion, this may explain the reduced toxicity in the homozygous compared to heterozygous mice.

In conclusion, hypoalbuminemia decreases the half-life of OTA in blood because of increased hepatocellular uptake and canalicular secretion in the liver and higher rates of glomerular filtration. This mechanism may be relevant for hypoalbuminemic patients exposed to OTA or to other toxic compounds with high plasma albumin binding.

## Supplementary Information

Below is the link to the electronic supplementary material.

Supplementary file1 Intravital imaging of OTA transport in the liver of wild-type (A) and albumin knockout heterozygous (B) and homozygous (C) mice. Blue: OTA; red: TMRE. The videos correspond to figure 2 of the main manuscript. Scale bars: 50 µm (M4V 38724 KB)

Supplementary file2 (M4V 38829 KB)

Supplementary file3 (M4V 42467 KB)

Supplementary file4 Intravital imaging of OTA transport in the kidneys of wild-type (A) and albumin knockout heterozygous (B) and homozygous (C) mice. Blue: OTA; red: TMRE. The videos correspond to figure 3 of the main manuscript. Scale bars: 50 µm. (M4V 11445 KB)

Supplementary file5 (M4V 13386 KB)

Supplementary file6 (M4V 11916 KB)

## Abbreviations

ACLD: Advanced chronic liver disease
ALT: Alanine transaminase
AST: Aspartate transaminase
BUN: Blood urea nitrogen
HA: Hypoalbuminemia
LD_50_: Median lethal dose
OTA: Ochratoxin A
OATs: Organic anion transporters
OATPs: Organic anion-transporting polypeptides
Mrp2: Multidrug resistance-associated protein 2
BCRP: Breast cancer resistance protein
NPT4: Na + -dependent phosphate transporter
TMRE: Tetramethylrhodamine-ethyl ester

## References

[CR1] Anzai N, Jutabha P, Endou H (2010). Molecular mechanism of ochratoxin a transport in the kidney. Toxins (basel).

[CR2] Balcar L, Semmler G, Pomej K (2021). Patterns of acute decompensation in hospitalized patients with cirrhosis and course of acute-on-chronic liver failure. United Eur Gastroenterol J.

[CR3] Bendele AM, Carlton WW, Krogh P, Lillehoj EB (1985). Ochratoxin A carcinogenesis in the (C57BL/6J X C3H)F1 mouse. J Natl Cancer Inst.

[CR4] Berg S, Kutra D, Kroeger T (2019). Ilastik: interactive machine learning for (bio)image analysis. Nat Methods.

[CR5] Campos G, Schmidt-Heck W, De Smedt J (2020). Inflammation-associated suppression of metabolic gene networks in acute and chronic liver disease. Arch Toxicol.

[CR6] Costa D, Simbrunner B, Jachs M (2021). Systemic inflammation increases across distinct stages of advanced chronic liver disease and correlates with decompensation and mortality. J Hepatol.

[CR7] Duarte SC, Pena A, Lino CM (2011). Human ochratoxin a biomarkers–from exposure to effect. Crit Rev Toxicol.

[CR8] EFSA (2020). Scientific opinion on the risks to public health related to the presence of ochratoxin A in food. EFSA J.

[CR9] Fanali G, di Masi A, Trezza V, Marino M, Fasano M, Ascenzi P (2012). Human serum albumin: from bench to bedside. Mol Aspects Med.

[CR10] George B, You D, Joy MS, Aleksunes LM (2017). Xenobiotic transporters and kidney injury. Adv Drug Deliv Rev.

[CR11] Ghallab A, Hofmann U, Sezgin S (2019). Bile microinfarcts in cholestasis are initiated by rupture of the apical hepatocyte membrane and cause shunting of bile to sinusoidal blood. Hepatology.

[CR12] Ghallab A, Myllys M, Holland CH (2019). Influence of liver fibrosis on lobular zonation. Cells.

[CR13] Ghallab A, Hassan R, Myllys M (2021). Subcellular spatio-temporal intravital kinetics of aflatoxin B1 and ochratoxin A in liver and kidney. Arch Toxicol.

[CR14] Ghallab A, Myllys M, Friebel A (2021). Spatio-temporal multiscale analysis of western diet-fed mice reveals a translationally relevant sequence of events during NAFLD progression. Cells.

[CR15] Ghallab A, Hassan R, Hofmann U (2022). Interruption of bile acid uptake by hepatocytes after acetaminophen overdose ameliorates hepatotoxicity. J Hepatol.

[CR16] Gianmoena K, Gasparoni N, Jashari A (2021). Epigenomic and transcriptional profiling identifies impaired glyoxylate detoxification in NAFLD as a risk factor for hyperoxaluria. Cell Rep.

[CR17] Greenblatt DJ, Koch-Weser J (1974). Clinical toxicity of chlordiazepoxide and diazepam in relation to serum albumin concentration: a report from the Boston Collaborative Drug Surveillance Program. Eur J Clin Pharmacol.

[CR18] Haller C (2005). Hypoalbuminemia in renal failure: pathogenesis and therapeutic considerations. Kidney Blood Press Res.

[CR19] Hartl L, Jachs M, Desbalmes C (2021). The differential activation of cardiovascular hormones across distinct stages of portal hypertension predicts clinical outcomes. Hepatol Int.

[CR20] Hartl L, Jachs M, Simbrunner B (2021). Cirrhosis-associated RAS-inflammation-coagulation axis anomalies: parallels to severe COVID-19. J Pers Med.

[CR21] Hassan R (2016). Possibilities and limitations of intravital imaging. Excli J.

[CR22] Holland CH, Ramirez Flores RO, Myllys M (2022). Transcriptomic cross-species analysis of chronic liver disease reveals consistent regulation between humans and mice. Hepatol Commun.

[CR23] IARC (1993). Ochratoxin A. IARC Monogr Eval Carcinog Risks Hum.

[CR24] Kanisawa M, Suzuki S (1978). Induction of renal and hepatic tumors in mice by ochratoxin A, a mycotoxin. Gan.

[CR25] Koeppert S, Ghallab A, Peglow S (2021). Live imaging of calciprotein particle clearance and receptor mediated uptake: role of calciprotein monomers. Front Cell Dev Biol.

[CR26] Kontaxi M, Echkardt U, Hagenbuch B, Stieger B, Meier PJ, Petzinger E (1996). Uptake of the mycotoxin ochratoxin A in liver cells occurs via the cloned organic anion transporting polypeptide. J Pharmacol Exp Ther.

[CR27] Kumagai S (1985). Ochratoxin A: plasma concentration and excretion into bile and urine in albumin-deficient rats. Food Chem Toxicol.

[CR28] Mally A, Dekant W (2009). Mycotoxins and the kidney: modes of action for renal tumor formation by ochratoxin A in rodents. Mol Nutr Food Res.

[CR29] Merlot AM, Kalinowski DS, Richardson DR (2014). Unraveling the mysteries of serum albumin-more than just a serum protein. Front Physiol.

[CR30] Muñoz K, Cramer B, Dopstadt J, Humpf HU, Degen GH (2017). Evidence of ochratoxin A conjugates in urine samples from infants and adults. Mycotoxin Res.

[CR31] O'Brien E, Dietrich DR (2005). Ochratoxin A: the continuing enigma. Crit Rev Toxicol.

[CR32] Österreicher CH, Penz-Österreicher M, Grivennikov SI (2011). Fibroblast-specific protein 1 identifies an inflammatory subpopulation of macrophages in the liver. Proc Natl Acad Sci U S A.

[CR33] Perry JL, Christensen T, Goldsmith MR, Toone EJ, Beratan DN, Simon JD (2003). Binding of ochratoxin A to human serum albumin stabilized by a protein−ligand ion pair. J Phys Chem B.

[CR34] Phillips A, Shaper AG, Whincup PH (1989). Association between serum albumin and mortality from cardiovascular disease, cancer, and other causes. Lancet.

[CR35] Poor M, Kunsagi-Mate S, Czibulya Z (2013). Fluorescence spectroscopic investigation of competitive interactions between ochratoxin A and 13 drug molecules for binding to human serum albumin. Luminescence.

[CR36] Reif R, Ghallab A, Beattie L (2017). In vivo imaging of systemic transport and elimination of xenobiotics and endogenous molecules in mice. Arch Toxicol.

[CR37] Reiss SN, Buie LW, Adel N, Goldman DA, Devlin SM, Douer D (2016). Hypoalbuminemia is significantly associated with increased clearance time of high dose methotrexate in patients being treated for lymphoma or leukemia. Ann Hematol.

[CR38] Remetic J, Ghallab A, Hobloss Z (2022). Loss of bile salt export pump aggravates lipopolysaccharide-induced liver injury in mice due to impaired hepatic endotoxin clearance. Hepatology.

[CR39] Ringot D, Chango A, Schneider YJ, Larondelle Y (2006). Toxicokinetics and toxicodynamics of ochratoxin A, an update. Chem Biol Interact.

[CR40] Schenk A, Ghallab A, Hofmann U (2017). Physiologically-based modelling in mice suggests an aggravated loss of clearance capacity after toxic liver damage. Sci Rep.

[CR41] Schneider KM, Candels LS, Hov JR (2021). Gut microbiota depletion exacerbates cholestatic liver injury via loss of FXR signalling. Nat Metab.

[CR42] Schneider KM, Elfers C, Ghallab A (2021). Intestinal dysbiosis amplifies acetaminophen-induced acute liver injury. Cell Mol Gastroenterol Hepatol.

[CR43] Sezgin S, Hassan R, Zuhlke S (2018). Spatio-temporal visualization of the distribution of acetaminophen as well as its metabolites and adducts in mouse livers by MALDI MSI. Arch Toxicol.

[CR44] Soeters PB, Wolfe RR, Shenkin A (2019). Hypoalbuminemia: pathogenesis and clinical significance. JPEN J Parenter Enteral Nutr.

[CR45] Sueck F, Poor M, Faisal Z (2018). Interaction of ochratoxin A and its thermal degradation product 2'R-ochratoxin A with human serum albumin. Toxins (basel).

[CR46] Sueck F, Specht J, Cramer B, Humpf HU (2020). Identification of ochratoxin-N-acetyl-L-cysteine as a new ochratoxin A metabolite and potential biomarker in human urine. Mycotoxin Res.

[CR47] Tao Y, Xie S, Xu F (2018). Ochratoxin A: toxicity, oxidative stress and metabolism. Food Chem Toxicol.

[CR48] Thévenaz P, Ruttimann UE, Unser M (1998). A pyramid approach to subpixel registration based on intensity. IEEE Trans Image Process: Publ IEEE Signal Process Soc.

[CR49] Vallner JJ (1977). Binding of drugs by albumin and plasma protein. J Pharm Sci.

[CR50] Vartak N, Guenther G, Joly F (2021). Intravital dynamic and correlative imaging of mouse livers reveals diffusion-dominated canalicular and flow-augmented ductular bile flux. Hepatology.

